# The Deciduous Teeth and Their Treatment—Causes of Irregularity of the Permanent Teeth, Etc.

**Published:** 1874-01

**Authors:** H. L. Sage


### THE DECIDUOUS TEETH AND THEIR TREATMENT—CAUSES OF IRREGULARITY OF THE PERMANENT TEETH, ETC.

BY H. L. SAGE, D. D. S.

The functions of the deciduous teeth, their relative value, physiologically, their care during the period allotted for their retention, their treatment when diseased, the effect of their premature loss, etc.,—are questions which have not received the attention due to their significance; for the advent of the incoming permanent teeth, and the necessary practice required for their maintenance in healthfulness and beauty, have seemingly left the less important consideration, what kind and degree of attention shall be directed to the temporary teeth, in the background. When dentists have practically treated the matter as one of secondary importance, or to be viewed only in connection with the influence which their premature loss might have in causing a contraction or preventing the full development of the jaw, and thus producing irregularity of the permanent teeth,—their value as conservators of health, being hardly if at all considered,—how can it be expected that parents will realize their value, or the importance of their preservation?

The deciduous teeth have, for the most part, received attention only when the sufferings of the child have rendered palliative treatment necessary, preventive treatment by filling, etc., being comparatively a rare procedure. The dernier resort, extraction, has been a not uncommon method, with many, of ridding one's self of the unwelcome little patient,—unwelcome so far as the necessity of conservative treatment would make it,—while, with the larger number, the matter has been considered in connection with extraction, i. e., with reference to the influence which the unseasonable loss of one or more of those teeth would have in checking the growth and expansion, or in causing contraction of the jaw, and upon the state of the future permanent teeth, as regards their position in the arch, their liability to caries, etc.: which, so far as it goes, is quite proper; but the value as conservators of health, and to this end their retention, by the prevention or checking of caries, and their maintenance in a healthful condition, should excite fully as much interest and induce as much effort as the other consideration, taking into account the ten or more years of early life in which important functions are to be materially aided by their preservation.

We cannot fully realize the effects—are ignorant in a measure, of the consequences, direct or indirect, of the premature loss of the deciduous teeth upon the future health and comfort of the child. How much of physical suffering may be entailed, to what degree the features may be robbed of their beauty, how greatly the functions of the digestive organs may be impaired, and nutrition checked by their premature removal, or in what degree the nervous system may suffer by the pain consequent upon a neglect of these teeth, cannot be a matter either of precognition or of after knowledge even.

One night of suffering may lay the foundation for disease; but more serious may be the consequences, when successive days and nights of odontalgic pains are suffered to elapse without mitigation,—together with the tenderness of exposed pulps and abscessed roots, which prevent perfect mastication of the food, and impair the digestive and assimilative functions. In short, the welfare and health of the individual in after life may be seriously compromised by a neglect of that prophylactic treatment which these teeth call for in the majority of cases, but which call is unheeded, except in a comparatively few instances.

If the child happens to be possessed of a delicate organization, especially, the dangers resulting from neglect are more imminent and serious. Certainly, during the formative period of infancy and childhood, whatever tends to prevent the development of a perfect organism should be avoided. Viewed in this light, is not the preservation of the deciduous teeth during the allotted time of as much relative importance as that of the permanent? But parents have not been educated to so regard them, and why is it that many dentists *practically* ignore the matter?

Probably, to state one reason, because of the difficulty and tediousness of operations upon children's teeth, and the patience and self-sacrifice required of a dentist who would discharge his duty in this respect. Aside from this, the confidence of the child must, or should, be obtained, by the successive tedious steps and various arts of persuasive power which must be brought into requisition ; together with such gentle manipulation as shall involve a greater sacrifice of time, and wear and tear of mind and body, than is necessary in the case of older patients.

Again : these operations do not directly pay, pecuniarily ; for most parents are not only unwilling to compensate the dentist for the *extra* time and labor required for operations on children's teeth, or to incur much or any expense, in order to preserve organs that are so soon to be cast off and replaced by more enduring structures. They view it only in connection with the temporary annoyance which aching teeth may cause them, or from a purely sympathetic feeling, which renders them unwilling to see the child suffer ; for they have not been taught how much depends upon thorough care and preventive treatment with these teeth, and the checking of caries before it has made sufficient progress to entail serious consequences.

Most writers have been wont to discourse upon the premature loss of one or more of the deciduous teeth, only as regards the effect upon the development of the maxilla, and the influence upon the permanent teeth in relation to their position in the arch, or, in other words, the production of irregularities. While most have maintained that contraction is consequent upon their removal before the time for the appearance of the teeth of replacement, a minority hold precisely adverse views.

A late writer in one of our dental journals—a prominent and able practitioner of many years experience—maintains that, so far as contraction is concerned, the premature loss of the deciduous teeth has no influence in the matter. He entirely ignores the question as to the effect in other directions, both physiologically and pathologically ; from which we may infer that he attaches no importance thereto, especially as he intimates that no harm, but rather benefit, will result from the removal of decayed and troublesome deciduous teeth ;—which idea he has practically carried out in his own family, thus showing his faith by his works.

It would be well for the profession and the public, were the question, "Does the premature loss of the deciduous teeth cause contraction of the jaws ?" definitely settled, and a more thorough and universal knowledge had of the growth and development of the maxilla, and the anatomical and physiological relations of it with the teeth therein implanted, in connection with the processes of first and second dentition ; and their influence upou the normal and abnormal development of the jaws, and proper relative position of the teeth to each other, as connected with symmetry of features, beauty of expression, perfection of articulation, and normal antagonisms of the teeth ; its influence upon the vocal powers, etc., etc.

Of course, every dentist cannot be, or, at least, has not the facilities, time, pecuniary means, natural ability or necessary physical vigor to become a thorough anatomist and microscopist ; consequently, light must be gained from the recorded and uttered observations of leading minds, who have made these things a special and life-long study. But, as such men hold divers diverse views, and opinions directly opposite, we can without egotism or presumption, after or while summing up the opinions of those savans, advance our own views or suggestions ; or, at least, propound inquiries, with a certain degree of propriety, which shall afford a basis for investigation and discussion ; and that, too, with the laudable object of doing the greatest amount of good of which we are capable, in our should-be-beneficent calling. Therefore the question heading this paper, with others emanating from it, has been selected. But, first, let us bring together some of the views of a few eminent writers, concerning the influence of the premature removal of the deciduous teeth in producing imperfectly developed jaws and irregularly positioned permanent teeth—for we shall find that it is only in this connection, that most of them refer to the unseasonable loss of the former.

Tomes, in his "Dental Surgery," says: "It has been usual to assume that the premature extraction of the temporary teeth occasions contraction of the jaws; but I do not think that any anatomical facts can be brought forward in support of the supposition. If a temporary tooth be removed, the crowns of the contiguous teeth may lean towards each other and give the appearance of contraction; but it does not really involve a diminished size of that part of the jaw from which the tooth was lost."

He further says: "Subsequently, however, there may be some amount of practical inconvenience, resulting from the premature removal of the deciduous teeth; but it is altogether independent of contraction of the jaw."

He terms it merely an "inconvenience," the direct or indirect effect upon the health being ignored. But we are wont to consider that improper mastication of the food is something more than an inconvenience, when dependent on the loss of *adult* teeth, and often produces disordered stomachs, and lays the foundation for dyspepsia and other diseases—why not in the case of the child?

Who has not observed marked improvement in the health of certain edentulous individuals, after having their losses repaired by serviceable dental substitutes?

We may infer, from Mr. Tomes' suggestions, that only the crown of a tooth may change position after losing the support which contiguous teeth may afford, and that contraction of the jaw is thus only apparent, and not real. But if the sixth-year molars, for example, are extracted at an early age, though the space from which they were taken will usually be occupied by the teeth anterior and posterior thereto, we will observe that, though in some cases the crowns only of the posterior or second molars will lean forward, in others, and the greater number, the entire tooth will have been moved in the same direction, and the crown thus continue to maintain an upright or perpendicular position—and in many instances, after the permanent teeth have all appeared, the jaw will yet be filled by the fourteen remaining teeth, showing that, had the sixth-year molars been retained, the jaw must have attained greater dimensions in order to give them room, or a crowded and irregular denture must have been the result.

It is not probable that, in all cases, the growth and expansion of the jaw would have kept pace with the amount of room required for the full number of erupted and erupting permanent teeth—at least it does not. We often observe the bicuspids out of position—standing either out or in. Though it is true that this is often caused by the presence of persistent temporary molars, yet it would seem reasonable to suppose that, as when the sixth-year molars are lost in childhood, the posterior teeth crowd forward, so, if the temporary second molars are prematurely removed, the sixth-year molars, by crowding against and overlapping the crowns of the incoming second bicuspids, might cause the latter to appear irregularly; and that this lateral and coronal pressure might extend to, and influence the position of the first bicuspids and tne canines as well.

If the theory, that the presence in the alveoli of the crowns of the permanent teeth serves to prevent contraction, even though the deciduous be entirely lost, were true, it might not be tenable, so far as the untimely loss of the eight temporary molars is concerned; for these being replaced by bicuspids, —teeth which are smaller,—the presence of the latter would not be likely to prevent the springing together of those portions of the jaw in which they were implanted, and from which the larger temporary molars had been removed. The time of removal would, of course, influence the extent or degree of contraction.

Prof. Garretson thus comments upon the process of development, in which he intimates that contraction is produced by the premature removal of the deciduous teeth: "The deciduous dental arch is filled, as we are all aware, completely by its ten teeth. The second or permanent set is to comprise in number sixteen, and each tooth certainly quite as large again as its predecessor. This increase in number and size of the teeth, it is evident, must be provided for in an enlargement of the alveolar arch. This provision is always attempted by nature in the process described by the physiologist as the elongatory. I will illustrate this process of maxillary enlargement, by considering the ten milk teeth as so many wedges placed in a springy arch. This arch it is designed to lengthen by additions to either end. If, now, these wedges should be removed before others were ready to take their places, it is evident that the elongation, being made at the ends, would, to a greater or less extent, be counterbalanced by the springing together of the parts at the sites of the removed wedges. The process of maxillary, or rather alveolar absorption, is truly represented by this retraction of the arch, at least of its alveolar face."

He also adds in a foot-note: "Prof. Gross, and Mr. Tomes also, in his last book denies this position; why, I know not. With the greatest respect for the opinion of these gentlemen, it is my experience that they are wrong."

The following quotation from Prof. Harris' work also affords deductions that seem reasonable. After stating that "after the completion of first dentition, that part of the alveolar border occupied by the first set of teeth augments in dimensions but very little," (meaning, perhaps, until the permanent teeth come to arrange themselves in the arch,) and that, during the continuance of the temporary, the increase is chiefly confined to the back part of the jaw, he cites the views of Delabarre, with which he coincides in this respect: "that the dimensions of the alveolar arch may be increased by pressure upon the teeth, from behind forwards"—referring to the mechanical influence of second dentition. But may not this pressure sometimes give rise to irregularities as well?

He also says: "The ten anterior permanent teeth occupy a somewhat larger space than that taken up by the temporary ones which preceded them, and were there no increase in the size of this portion of the arch, the regularity of this arrangement would be more or less disturbed. To prevent this, a slight increase is necessary, but the dimensions of that portion of the alveolar border occupied by the temporary teeth is not materially increased until these teeth are shed, and then, as those of replacement come forward to take their places, they arrange themselves in a somewhat larger arch;" and thus,

"the size of the alveolar border is augmented, forming a new ridge slightly larger than the first." "But there is not always an increase in the anterior part of the jaws; on the contrary, the premature loss of one or more of the temporary teeth often occasions a contraction that frequently causes irregularity of the permanent set," etc.

Thus it seems that, after the completion of first dentition, that portion of the jaw proper occupied by the ten deciduous teeth does not usually, as it is maintained, augment materially in size, the alveolar face being the part undergoing the most change, by or during the process of second dentition;—and although the permanent incisors, canines and bicuspids, aside from the molars, require a larger space than was requisite for the temporary, much or most of the additional room must be made up by the elongation at the ends; but as this is not always sufficient, the result is an irregular denture.

So, too, the jaw is, at this age, when nutrition is normal, undergoing other changes, i. e., becoming harder and denser by the ossific matter which is deposited for the nourishment and building up of bone-tissue;—and, if so, why may not a retrograde movement or process also occur by any disturbing agency? Why may not nutrition be checked by a cessation of functional activity in those portions of the arch in which the loss of teeth may have occurred? For they would lack the stimulus (work) which other portions would receive, where the teeth were not absent. Why may not lateral pressure influence contraction, or, rather, cause the incoming teeth against which pressure was directed to crowd upon each other, and thus become irregular in position? Besides, though this por- the jaw may have attained full size, it may not have reached full density and, perhaps, not full toughness.

Muscles become useless, flabby, and diminish in size and strength by remaining idle. Might not this have an influence in causing a diminution and waste or in checking the growth of the jaw proper, at the sites of the prematurely removed teeth, as well as prevent the full development of the alveolar face? and though growing organs influence the growth and expansion of the parts about them, as is the case with the erupting permanent teeth and their alveoli, it might not entirely compensate for the loss caused by the previous failure of the parts in which the loss occurred to perform their functions. Full development may thus be prevented.

As stated by Mr. Tomes, "arrested growth of the jaw, at any period prior to the eruption of the permanent teeth, may produce lateral contraction of the dental arch ; or the persistence of the temporary teeth may turn the bicuspids inwards. Although not a common cause, cases may be found in which disease in the temporary molars, and subsequent alveolar abscess, have occasioned the displacement of the bicuspids."

If alveolar abscess may cause displacement, may it not also cause contraction ?

From a summing up of facts it would appear :

That the deciduous teeth occupy a space in the arch considerably smaller than the same number of the teeth of replacement ;

That the jaw proper, in its anterior portion, has attained nearly full size by the time the first teeth have all appeared, though it continues to augment transversely and perpendicularly a little, until the completion of the second dentition ;

That there is not always an increase in the anterior part of the jaws ;

That the elongation of the jaw at the ends is a provision of nature to afford room for the incoming first, second and third molars principally, and that the additional space gained by the elongatory process is not sufficient in many cases for this, as is well known from the many severe cases which appear during the eruption of the dentes sapientiæ.

From these facts, it would not be surprising did irregularities of position in the permanent teeth occur independently of the premature extraction of the deciduous.

It would also appear :

That mechanical pressure of the large grinding teeth from behind forwards might produce irregularity of the bicuspids, canines and incisors ;

That, in case of the untimely loss of the temporary molars, the sixth-year molars, by crowding against and overlapping the incoming bicuspids, might cause them to appear irregu-arly;

That the premature loss of the temporary molars might produce irregularity of the bicuspids, which, being smaller, could not, by their presence in the jaw, prevent a springing together of those portions of the arch in which they were implanted; just as removing a larger stone from an arch, and leaving a smaller one in its place, might produce its contraction or diminish its diameter;

That lateral pressure, or overlapping by the permanent incisors and bicuspids, against or on the canines, before the appearance of the latter, may often cause them to appear out of the arch, especially if the temporary canines are removed prematurely;

That the premature removal of one or more of the deciduous teeth removes the stimulus to growth and development (work) at the sites of the removed teeth, which was had in the active employment of that portion of the jaw in the mastication of the food and, might also result in the irregularity of the future permanent teeth; for the use of an organ induces its growth and expansion, and develops its power;

That this influence would extend to the corresponding teeth in the opposing jaw, from the loss of their antagonists;

That this might result also, if teeth were suffered to remain with exposed and tender pulps or abscessed roots, so as to prevent proper occlusion and mastication with these teeth:

We have kept out of view, in this connection, hereditary influences,—which are a frequent cause of dental irregularity and so acknowledged, and upon which it is therefore unnecessary to enlarge.

Concerning the importance of retaining the deciduous teeth during the allotted time, it would seem that it has not been overestimated.

As to the treatment, that is both prophylactic and mechanical. Cleanliness is as essential with these teeth as with the permanent, or nearly; and the first step should be, to inculcate and teach the use of the brush, and, when necessary, the removal by the dentist of the green stain or deposit which often adheres with such tenacity to children's teeth, and the polishing of the surfaces. The use of the brush should be taught as soon as the child has sufficient intelligence to learn it, and previous to that time, the duty should be performed for him.

Next, systematic examinations by the dentist should be had, and, where cavities have formed, the decay should be checked by filling; though, if it is in its incipiency, perhaps the treatment recommended by Dr. Arthur—that of filing—may be adopted with safety. If his system is applicable at all, it is in the treatment of incipient caries of the deciduous teeth, when situated proximally.

For fillings in these teeth, it is usually best, and even necessary, to insert a plastic material; as injury might be caused, not only to these, but the incoming permanent teeth, by the violence which might accompany the insertion of gold or tin, leaving out of view its (almost) impracticability. But it is presumed that no one attempts to fill the temporary teeth with materials requiring so much time, force and manipulation for their insertion as the latter, even could the control of the child be had sufficiently to permit it—which it is not ordinarily possible to obtain. And though such a thing has been accomplished, with very firm teeth, and in the case of a child whose confidence had been strengthened by familiarity with such things, and whose strong and robust organism permitted it and made it a pleasure to her,—it is very rarely feasible. Therefore, amalgam or Hill's stopping, preferably the former, when the conditions are such as to permit it, is the best for this purpose. When these are employed, and the filling is a proximal one, the permanent separation of the teeth with chisel or file is essential; for teeth filled with these materials ought never to be allowed to come together; for they should be rendered self-cleansing—as the materials themselves are not.

The sixth year molars, however, can be filled with gold, when the age, patience and endurance of the little patient is sufficient; but, if not, a temporary filling will answer, to be renewed from time to time, if necessary, and finally to be replaced by a more enduring one, when the child attains to greater strength of mind and body, so that he can not only bear the operation but appreciate its importance.

The little girl above referred to had an inferior molar filled with the oxy-chloride of zinc at the age of eighteen months, which was not long afterward renewed. Subsequently, one gold, one tin and two amalgam fillings were inserted—the inferior molars being the only teeth which were defective. This may have been caused by the frequent administration, for ulcerated sore throat, of an alkaline medicine, composed of the chlorate of potassa, bicarb. soda, etc. The other teeth are all perfect, free from stains and accretions, and beautiful in appearance.

The child, now over five and one-half years of age, is, and has been very healthy, with the exception of the attacks before referred to, which have ceased to recur. It would appear that this alkaline preparation may have had some effect in producing caries of these particular teeth, which were the most exposed to this action. The child would beg to have her teeth filled, and her behavior during the operation was very quiet and commendable, as well as an example worthy of imitation by some "children of a larger growth."

But, to revert to the subject under consideration, what treatment shall we pursue with exposed deciduous pulps?

When, from this cause, it becomes necessary to destroy a deciduous pulp, it may be done, by using the requisite caution, with the materials usually employed for the purpose; for, owing to the large pulps and foramina of these teeth, and to the fact that absorption of the roots may have taken place to a greater or less degree, care in this respect would be essential, guarding us, as Prof. Garretson remarks, "against a treatment in aching milk-teeth, at certain periods, which would be most applicable in others To judge of the condition of the foramina of the first teeth, we compare them with the periods of eruption of the second, recognizing that the enlargement or absorption corresponds with such advance in the permanent."

Referring to the employment of arsenic, he says: "In referring to the physiological changes constantly in progress in young teeth, and particularly the deciduous set, the inadvisability of arsenic as an application is at once made evident. Here the foramina are in various conditions of enlargement, and applications of such character would, of course, pass through these to the parts beyond."

But if the theory of Prof. Flagg, concerning the action of arsenious acid, is true, it may be a matter of some doubt whether there would be danger from its application to *deciduous* pulps, by its liability to pass through the foramina to the adjacent parts.

According to this observer, it first occasions a determination of blood to the pulp; secondly, congestion; and thirdly, inflammation and death of the portion nearest to the destructive agent. This increased circulation produces congestion, and the latter strangulation, "preventing first the egress and secondly the ingress of blood, and stopping all functional action." Thus, in adult teeth, this would prevent the arsenic from passing through the apicial foramen—and why might not the congestion also have the same effect in the larger pulps of the deciduous teeth, as it would prevent all circulation—which latter would be necessary to carry it into the adjacent tissues?

It would seem that the danger would be very small, excepting, perhaps, where much absorption of the root had taken place; and then the time would have quite or nearly arrived for the appearance of the teeth of replacement, when the destruction of the pulp could hardly be necessary. But it will usually be found that, when a child presents with a tooth aching from an exposed pulp, if the latter is carefully capped with the oxy-chloride of zinc, it will not only cause a cessation of pain, but the tooth will remain comfortable thereafter judging from cases in my own practice.

If thought best, the capping may be protected, and the tooth saved, by filling over the oxychloride with amalgam.

Abscessed deciduous teeth, with fistulas, may be sometimes treated to advantage, the disease removed, and the teeth filled.

Where the abscess has caused such destruction of alveolus as to lay bare the apex of the root, and it produces irritation of the lip or cheek, the root may be amputated in order to remove the protruding portion—which will at once remove the irritation and retain the tooth in place ; for it often happens that the incisor teeth especially come to us at a very early age, and long before the time for shedding, in this condition, when it would be clearly unwise to extract them, for the reason that contraction of the arch and irregularity of the permanent set might be the result of such practice, even were the first teeth rendered useless for other purposes. Of course, there are aggravated cases, in which temporary teeth are presented to our notice, when premature extraction becomes imperative —premature as regards the retention of organs whose presence is more injurious than their absence.

It is often distressing to observe the remnants of deciduous teeth, the temporary molars for instance, bereft almost or quite of their crowns and alveoli, several years before the time for the appearance of the bicuspids ; and when it becomes necessary to extract them, the conviction almost unavoidably arises, that their untimely loss, though perhaps a necessity, must be attended with injury,—that the jaw may suffer in its development, the teeth of replacement appear irregular or defective in structure and conformation, while the child, from the want of ability to properly masticate its food, and that strength and tone of system which accompany well-developed jaws, and sound, well-formed or well-cared-for deciduous teeth, may suffer permanent and lasting injury.

But enough. This matter, the treatment of the deciduous teeth, as herein set forth, is merely a general view of the subject. Many points yet remain untouched, to which attention might be directed with profit ; but the intention of this paper has in a measure been fulfilled.

Let the good work go on.

